# Case Report: Occupational bullous dermatosis: first report of skin lesions induced by PVC welding fume in a manufacturing worker

**DOI:** 10.3389/fimmu.2025.1648829

**Published:** 2025-09-26

**Authors:** Angela Stufano, Piero Lovreglio, Gerardo Cazzato, Paolo Danza, Riccardo Ravallese, Nicoletta Cassano, Gino Antonio Vena, Francesca Ambrogio, Benedetta Tirone, Caterina Foti

**Affiliations:** ^1^ Department of Medical and Surgical Science, University of Foggia, Foggia, Italy; ^2^ Interdisciplinary Department of Medicine, Section of Occupational Medicine, University of Bari, Bari, Italy; ^3^ Department of Precision and Regenerative Medicine and Ionian Area, Section of Molecular Pathology, University of Bari, Bari, Italy; ^4^ Department of Precision and Regenerative Medicine and Ionian Area, Dermatological Clinic, University of Bari, Bari, Italy

**Keywords:** occupational dermatoses, bullous pemphigoid, bullous dermatosis, PVC welding fumes, chemical exposure

## Abstract

**Purpose:**

Bullous dermatoses encompass a group of disorders marked by blister formation on the skin and/or mucosa with diverse etiologies. This case report aims to describe a rare occupationally induced bullous dermatosis in a worker exposed to polyvinyl chloride (PVC) welding fumes, and to highlight the need for improved protective measures in industrial environments.

**Methods:**

A 48-year-old male employed in a PVC manufacturing plant developed recurrent bullous skin lesions on the hands, face, and neck after operating a PVC film-welding machine without personal protective equipment. Clinical evaluation was supported by histopathology, direct and indirect immunofluorescence, serologic testing (ELISA for anti-desmoglein-1, anti-desmoglein-3, BP230, and BP180), and a porphyrin screen. Patch tests and autoimmunity screening were also performed.

**Results:**

Direct immunofluorescence revealed linear C3 and IgG deposits along the basement membrane zone, consistent with a bullous pemphigoid-like pattern, while other autoimmunity markers were negative. Complete remission of lesions occurred after cessation of exposure and job reassignment, without the need for ongoing pharmacologic treatment. A clear “stop-restart” relationship between exposure and symptoms supported a causal association.

**Conclusion:**

This report describes the first documented case of bullous dermatosis triggered by occupational exposure to PVC welding fumes. The findings emphasize the relevance of occupational assessment in unusual dermatologic presentations and support the implementation of adequate protective measures and exposure monitoring in high-risk settings.

## Introduction

Bullous dermatoses represent a group of disorders with varying etiopathogenesis, characterized primarily by the formation of blisters on the skin and/or mucous membranes, which often progress to erosions following rupture. Contact of the skin with drugs or chemicals can induce cutaneous reactions characterized by bullous lesions (1–3). However, the role of toxicants as causative agents in bullous skin disorders remains poorly understood and insufficiently documented.

Polyvinyl chloride (PVC), one of the most widely used synthetic polymers, is synthesized through the polymerization of vinyl chloride monomer. As the third most extensively produced synthetic polymer globally, PVC’s composition includes 57% chlorine and 43% carbon and hydrogen atoms upon completion of synthesis (4).

While PVC is valued for its versatility, it is inherently unstable under light and heat exposure, degrading with the release of hydrochloric acid (HCl). To address this, various stabilizing additives are incorporated, such as organometallic tin compounds, cadmium carboxylates, and metal salts of fatty acids. Other additives like lubricants (e.g., metal soaps, paraffinic waxes, and silicone) and flame retardants (e.g., zinc stannate, zinc borate, and antimony trioxide) enhance PVC’s processability and flame resistance. These additives are blended into the polymer in the form of powders, granules, or dry blends, tailored for specific applications (4).

The versatility of PVC is further enhanced by the addition of plasticizers, which transform the rigid polymer into flexible, moldable plasticized PVC. High molecular weight phthalates or phthalic acid esters are commonly used plasticizers, introduced based on processing needs. Plasticized PVC can then be shaped using techniques like hot pressing, extrusion, calendering, or liquefaction (4).

Under standard conditions, plasticized PVC is generally considered stable and safe. However, high temperatures pose significant risks. When heated or burned, plasticized PVC can release harmful substances such as HCl, dioxins, and furans, largely due to its chlorine content. Additionally, the variety of additives used in its production may contribute to health risks, both at room and high temperatures. These hazards underscore the critical need for comprehensive risk assessments, particularly for workers involved in PVC manufacturing or handling PVC-based materials. Occupational medicine plays a key role in addressing these risks.

Despite the well-documented association between PVC fumes and respiratory conditions like asthma (5), research into its effects on skin diseases remains limited. This knowledge gap is partly due to the difficulty of identifying specific toxic agents among the numerous additives in PVC formulations (5, 6).

## Case report

In February 2022, a 48-year-old Caucasian man was referred to the Dermatology Department of Bari University Hospital (Apulia, Italy) due to recurrent bullous lesions predominantly involving uncovered areas. The patient was an ex-smoker (approximately 15 cigarettes daily until 2018) and reported a personal history of atopy, including childhood atopic dermatitis, allergic rhino-conjunctivitis, and allergic asthma diagnosed in 2018. Skin prick tests had previously shown sensitivity to cat hair and dander, house dust mite, and grass pollen. His asthma was treated with as-needed inhaled salbutamol, supplemented with a combination of beclomethasone dipropionate and formoterol fumarate between March and June 2021. He denied any other drug therapy, as well as any personal or family history of bullous skin conditions.

The patient had been employed since 1993 in a PVC outdoor furniture manufacturing plant, operating a PVC sheet cutter. He reported no symptoms until September 2021, when he developed scattered blisters, accompanied by itching, especially on his face, neck and hands. These lesions appeared a few days after being assigned to operate a PVC film-welding machine, which utilized heated metal plates to join PVC flexible film. During this process, fumes were reportedly emitted, and the patient confirmed no use of personal protective equipment (PPE) and the absence of local exhaust ventilation.

In November 2021, following a worsening of his skin lesions, the patient underwent a 10-day course of oral prednisone (25 mg daily) on the advice of a dermatologist, experiencing a partial improvement of lesions in most areas except for the dorsal aspect of his hands. By mid-December 2021, during a holiday break, the lesions fully resolved, leaving only pruritus. A subsequent two-week sick leave in late December led to the complete remission of both skin lesions and itching.

**Figure 1:**
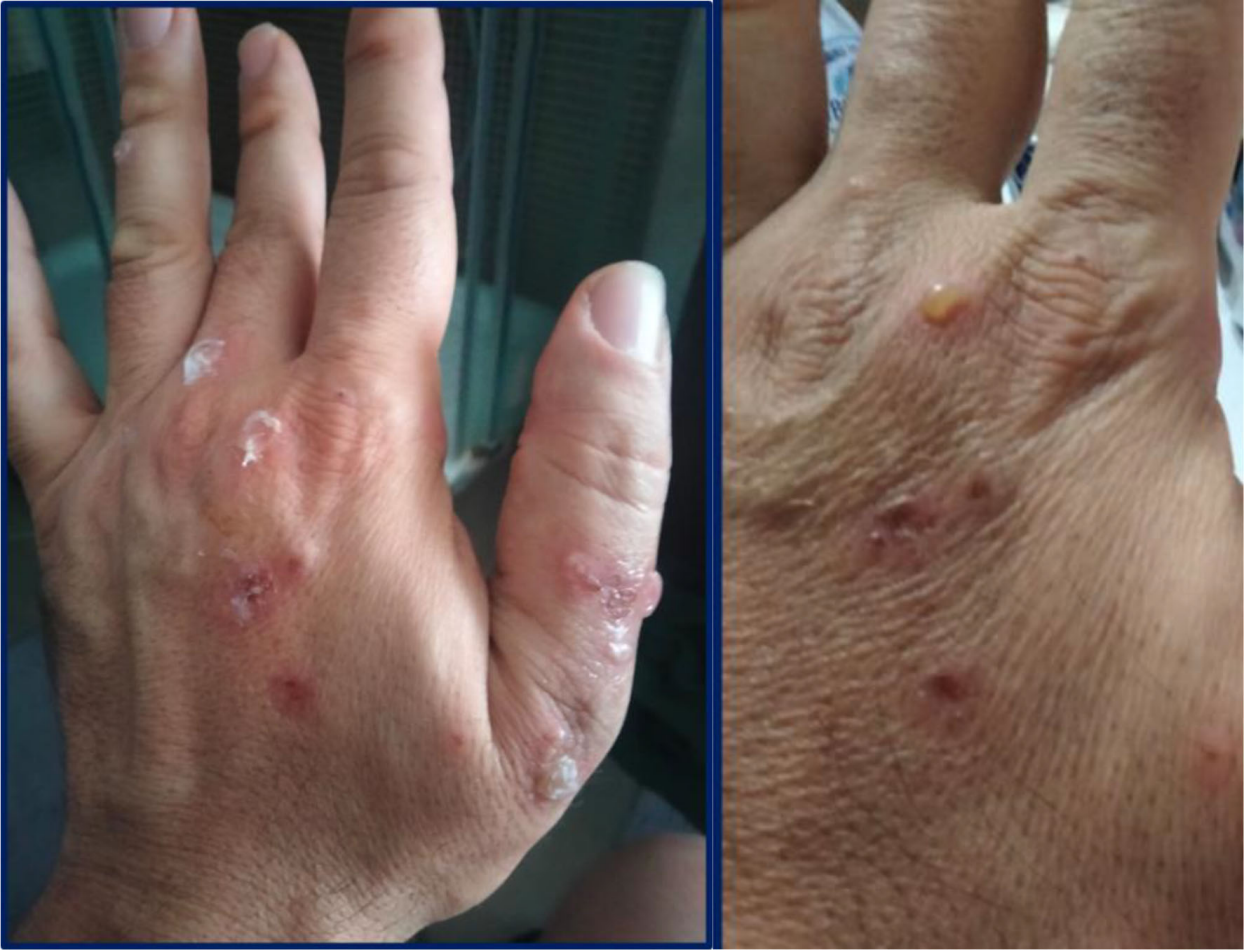
Fluid-filled tense blisters and superficial crusted erosions on the dorsal aspect of the right hand, with surrounding erythema.

**Figure 2:**
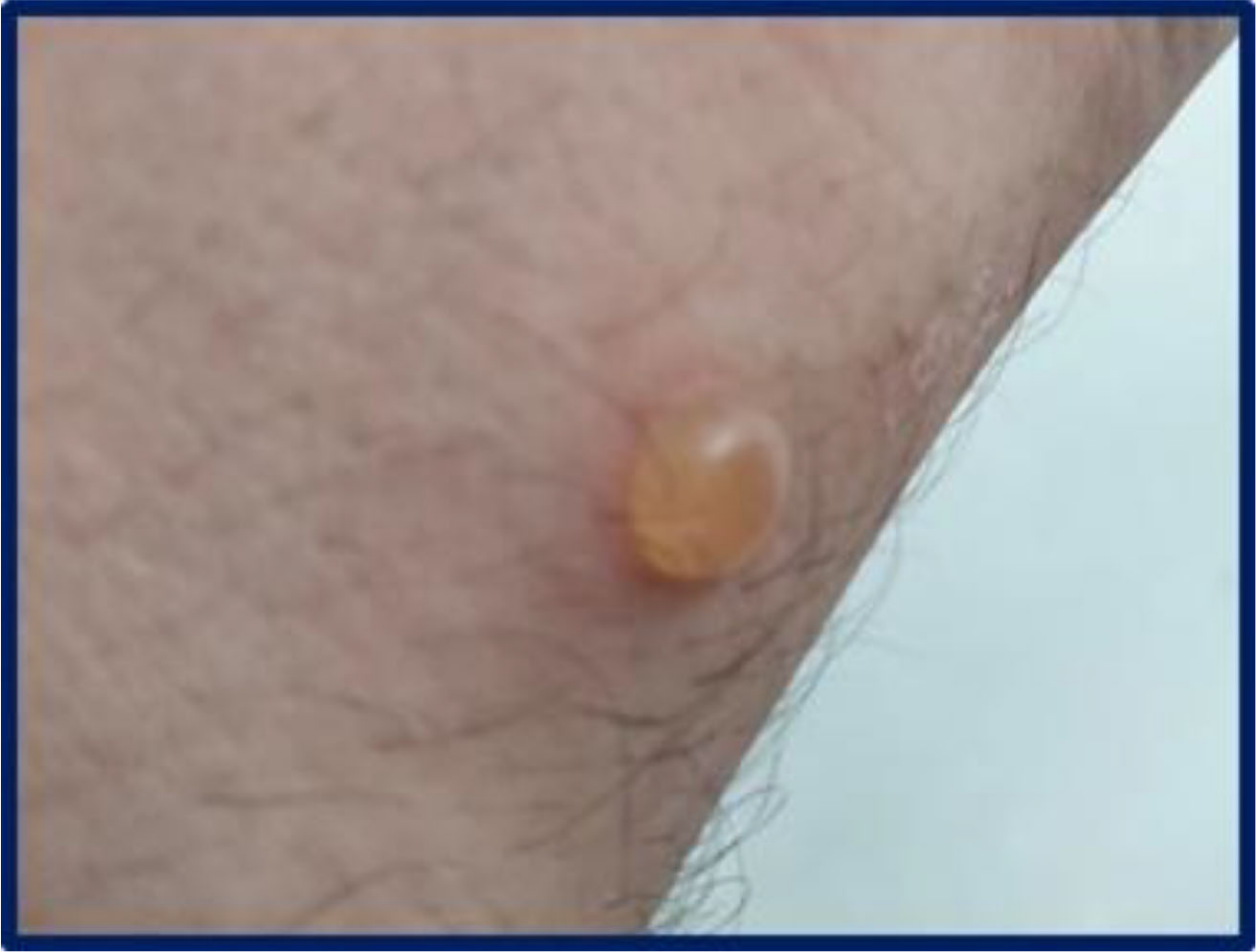
Single, well-demarcated tense bullous lesion containing clear fluid on the volar side of the right wrist.

In January 2022, upon resuming his duties at the same PVC film-welding workstation, the patient developed larger and more numerous bullous lesions, accompanied by more severe itching. These lesions predominantly affected the dorsal aspect of his hands and wrists (**Figures 1**, **2**), forearms, and neck, prompting his referral to Bari University Hospital in February. On examination, he was in good general health and afebrile. Mucosal involvement was absent and Nikolsky sign was not elicitable.

Suspecting an autoimmune bullous dermatosis, skin biopsies were performed for histopathologic evaluation and for direct immunofluorescence (DIF) studies. Histopathology revealed an infra-epidermal detachment with accumulation of fibrinous material and leucocytes, including eosinophils with focal aggregates of neutrophils, forming blisters with secondary re-epithelialization, mimicking pemphigus. Necrosis of keratinocytes in superficial epidermal layers with signs of eosinophilic spongiosis was also observed. A perivascular and periadnexal infiltrate in the superficial and mid dermis, predominantly made of lymphocytes with some eosinophils, was present.

DIF of the perilesional skin demonstrated linear deposits of C3 and IgG along the basement membrane zone (BMZ), consistent with a diagnosis of bullous pemphigoid (BP). However, indirect immunofluorescence using monkey esophagus as substrate excluded the presence of circulating autoantibodies (intercellular space and BMZ staining patterns).

Laboratory examinations, including blood count, serum glucose, renal and liver function tests, revealed normal findings with the only exception of elevated total IgE (2030 IU/mL; reference values <100 IU/mL). A comprehensive porphyrin screen ruled out porphyria. In addition, enzyme-linked immunosorbent assay (ELISA) for detection of anti-desmoglein-1, anti-desmoglein-3, anti-BP230 and anti-BP180 antibodies was negative. Negative results were also obtained for anti-nuclear antibodies, tissue transglutaminase autoantibodies (IgG and IgA) and antibodies against extractable nuclear antigens. An abdominal ultrasonography was normal. Patch tests using the SIDAPA (Società Italiana di Dermatologia Allergologica Professionale e Ambientale) baseline series yielded negative results.

While awaiting definitive results, the patient was reassigned to PVC packing and road haulage tasks. Following this change in his work activity, the patient reported complete remission of skin lesions at a six-month follow-up, without requiring any systemic or topical therapy.

## Discussion

This case report presents a manufacturing worker who developed bullous skin lesions a few days after exposure to PVC welding fumes. Although the diagnostic findings included atypical features, the results of DIF were suggestive of BP. BP is the most common autoimmune subepidermal blistering disorder and is characterized by the presence of tense blisters on erythematous or normal skin, although atypical presentations exist. The disease is linked to the production of autoantibodies targeting BP180 and BP230, two components of the hemidesmosome. BP has been associated with systemic or topical exposure to certain drugs (1, 7). Several features observed in this case, including the patient’s young age, prominent eosinophilic infiltration, and keratinocyte necrosis on histology, align with descriptions of drug-associated BP (7, 8).

Intraepidermal vesicles, although atypical for pemphigoid, have been reported in some cases of BP (9) and also in drug-associated BP (7). The possible presence of intraepidermal blisters in BP seems to be mostly related to re-epithelization and more rarely to spongiotic vesiculation (10, 11). The absence of subepidermal blisters, which are typical of pemphigoid, in our case was likely due to secondary re-epithelializationand signs of eosinophilic spongiosis were also detected on histopathology. Spongiotic pattern of BP without subepidermal clefting may mimic eczematous dermatitis or hypersensitivity reaction and has been described in urticarial forms of BP and in prodromal BP (12–14). Anti-BP180 and anti-BP230 autoantibodies, commonly assessed for BP diagnosis, were absent in this case. However, the absence of circulating autoantibodies does not rule out BP, as these autoantibodies may be undetectable in some cases, including atypical forms (15, 16).

As reviewed by Bağcı et al. (17), a subset of patients may lack detectable serum autoantibodies despite compatible clinical, histological, and direct immunofluorescence findings. Several mechanisms may explain seronegativity, including the presence of antibodies directed against atypical epitopes not covered by conventional assays, low autoantibody titers below detection thresholds, or technical limitations of the diagnostic tests. In our case, the absence of circulating autoantibodies may also reflect an atypical, possibly exposure-related variant of BP. Nonetheless, the combination of suggestive histopathology, positive direct immunofluorescence, and complete resolution of symptoms after exposure cessation supports the diagnosis of a seronegative occupational BP.

To our knowledge, this is the first reported case of a bullous disorder triggered by occupational exposure to PVC welding fumes.

The relationship between the skin lesions and occupational exposure was reinforced by a positive “stop-restart” test, with complete resolution of lesions after removal from the PVC welding workstation and no need for systemic therapy. This self-limited course mirrors that of drug-associated BP, which can resolve upon withdrawal of the offending agent (8).

PVC welding occurs at temperatures between 250 and 280°C, within the thermal range (225–475°C) where PVC degrades and releases volatile substances. These include hazardous additives such as plasticizers, stabilizers, lubricants, and flame retardants, which can emit toxic fumes under high heat. These conditions likely contributed to the patient’s exposure to airborne toxicants during the welding process. While we cannot quantify the exact duration or intensity of exposure, the description of the patient’s work environment—absence of PPE and local exhaust ventilation—supports the plausibility of a significant and repeated exposure. The lack of direct environmental measurements constitutes a limitation, yet the strong temporal association and reproducibility of symptom onset and resolution reinforce the suspected causal link.

The worker’s failure to use PPE during welding likely amplified exposure to these fumes. Although other employees performing the same task reported no dermatologic problems, individual factors, including the worker’s history of atopy, may have predisposed him to an exaggerated immune response. While allergic dermatitis from PVC products, such as gloves, has been reported previously (6), there are no prior documented cases of bullous dermatoses caused by PVC fumes.

Material Safety Data Sheets (MSDS) for PVC products are crucial for identifying potential hazards. However, in this case, the MSDS for the PVC material used provided only a summary of additive information, limiting the ability to pinpoint the specific substances released during welding. This incomplete documentation, noted in prior studies as a challenge in recognizing PVC-related toxicities (6), hindered the identification of the causative agent in this case.

A limitation of this case report is the lack of quantitative environmental exposure assessment, such as air sampling or analysis of fume composition. Although the strong temporal association and the stop-restart pattern support a causal link, the absence of direct exposure data limits the ability to establish a definitive mechanistic relationship. Future studies should incorporate detailed environmental monitoring to identify specific agents involved in similar cases. Another limitation of this report is the absence of immunoblot analysis, which could have helped detect circulating autoantibodies not identified by standard ELISA or indirect immunofluorescence techniques.

## Conclusion

This report underscores the potential for occupational exposure to PVC welding fumes to trigger bullous dermatoses in susceptible individuals. The absence of PPE and exposure to airborne toxicants likely contributed to the patient’s condition, while the complete resolution of symptoms upon removal from exposure highlights the role of occupational factors.

The case also emphasizes the need for detailed and transparent reporting of hazardous substances in industrial products through comprehensive MSDS documentation. Such measures would enhance risk assessment, enable targeted preventive strategies, and aid in identifying specific triggers in occupational health cases. Further research is needed to better understand the relationship between PVC exposure and bullous dermatoses, ensuring improved protection for workers and a reduced burden of occupational skin diseases.

## Data Availability

The raw data supporting the conclusions of this article will be made available by the authors, without undue reservation.

## References

[1] Vassileva S. Drug-induced pemphigoid: bullous and cicatricial. Clin Dermatol. (1998) 16:379–87. doi: 10.1016/s0738-081x(98)00008-x, PMID: 9642531

[2] Vogel TA Christoffers WA Engfeldt M Bruze M Coenraads PJ Schuttelaar ML. Severe bullous allergic contact dermatitis caused by glycidyl methacrylate and other acrylates. Contact Dermatitis. (2014) 71:247–9. doi: 10.1111/cod.12247, PMID: 25231388

[3] Tammaro A Adebanjo GAR Chello C Magri F Sernicola A Gagliostro N. Bullous dermatitis caused by common juniper. Contact Dermatitis. (2020) 83:529–31. doi: 10.1111/cod.13674, PMID: 32712995

[4] Höfer R. Polymers for a sustainable environment and green energy. Elsevier eBooks. Elsevier BV (2012) 10. doi: 10.1016/B978-0-444-53349-4.00301-0

[5] Jaakkola JJ Knight TL. The role of exposure to phthalates from polyvinyl chloride products in the development of asthma and allergies: a systematic review and meta-analysis. Environ Health Perspect. (2008) 116:845–53. doi: 10.1289/ehp.10846, PMID: 18629304 PMC2453150

[6] Park SG Lee EC Hong WK Song HJ Shin JH. A case of occupational allergic contact dermatitis due to PVC hose. . J Occup Health. (2008) 50:197–200. doi: 10.1539/joh.n7015, PMID: 18403872

[7] Verheyden MJ Bilgic A Murrell DF. A systematic review of drug-induced pemphigoid. Acta Derm Venereol. (2020) 100:adv00224. doi: 10.2340/00015555-3457, PMID: 32176310 PMC9207627

[8] Stavropoulos PG Soura E Antoniou C. Drug-induced pemphigoid: a review of the literature. J Eur Acad Dermatol Venereol. (2014) 28:1133–40. doi: 10.1111/jdv.12366, PMID: 24404939

[9] Alcalay J David M Ingber A Hazaz B Sandbank M. Bullous pemphigoid mimicking bullous erythema multiforme: an untoward side effect of penicillins. J Am Acad Dermatol. (1988) 18:345–9. doi: 10.1016/s0190-9622(88)70050-x, PMID: 2964460

[10] Joshi R. Spongiotic intra-epidermal blister: a pitfall in the histopathologic diagnosis of bullous pemphigoid. Indian J Dermatol. (2013) 58:410. doi: 10.4103/0019-5154.117355, PMID: 24082221 PMC3778816

[11] Batrani M Kubba A Kubba R. Incidental acantholysis in a case of bullous pemphigoid-A diagnostic quandary. J Cutan Pathol. (2025) 52:259–61. doi: 10.1111/cup.14724, PMID: 39313847

[12] Amber KT Valdebran M Kridin K Grando SA. The role of eosinophils in bullous pemphigoid: A developing model of eosinophil pathogenicity in mucocutaneous disease. Front Med (Lausanne). (2018) 5:201. doi: 10.3389/fmed.2018.00201, PMID: 30042946 PMC6048777

[13] Atteh G Cole EF Perricone AJ Feldman RJ. Bullous eczema presenting as bullous pemphigoid-like eruption: A case series. JAAD Case Rep. (2021) 10:34–7. doi: 10.1016/j.jdcr.2021.01.032, PMID: 33732844 PMC7941078

[14] Merton A Wu YH. Spongiform pemphigoid: A case series of an uncommon histopathologic pattern. J Cutan Pathol. (2020) 47:339–45. doi: 10.1111/cup.13627, PMID: 31837162

[15] Obijiofor CE Ogah O Anyanwu N Akoh CC Moshiri AS Culton DA. Insights into bullous pemphigoid: A comprehensive review of diagnostic modalities. JAAD Rev. (2025) 3:26–36. doi: 10.1016/j.jdrv.2024.11.004

[16] Liu Y Li L Xia Y. BP180 is critical in the autoimmunity of bullous pemphigoid. Front Immunol. (2017) 8:1752. doi: 10.3389/fimmu.2017.01752, PMID: 29276517 PMC5727044

[17] Bağcı IS Horváth ON Ruzicka T Sárdy M. Bullous pemphigoid. Autoimmun Rev. (2017) 16:445–55. doi: 10.1016/j.autrev.2017.03.010, PMID: 28286109

